# Functionalized SnO_2_ nanoparticles with gallic acid via green chemical approach for enhanced photocatalytic degradation of citalopram: synthesis, characterization and application to pharmaceutical wastewater treatment

**DOI:** 10.1007/s11356-022-22447-5

**Published:** 2022-08-15

**Authors:** Veronia S. Nazim, Ghada M. El-Sayed, Sawsan M. Amer, Ahmed H. Nadim

**Affiliations:** Analytical Chemistry Department, Faculty of Pharmacy, Cairo University, Kasr El-Aini st, Cairo, Egypt

**Keywords:** Pharmaceutical wastewater treatment, SnO_2_ nanoparticles, Gallic acid, Photocatalytic degradation, Cleaning validation, UV irradiation

## Abstract

Eco-friendly stannic oxide nanoparticles functionalized with gallic acid (SnO_2_/GA NP) were synthesized and employed as a novel photocatalyst for the degradation of citalopram, a commonly prescribed antidepressant drug. SnO_2_/GA NP were characterized using high-resolution transmission electron microscopy, Fourier transform infrared spectroscopy, Brunauer–Emmett–Teller measurements and X-ray diffraction. A validated RP-HPLC assay was developed to monitor citalopram concentration in the presence of its degradation products. Full factorial design (2^4^) was conducted to investigate the effect of irradiation time, pH, SnO_2_/GA NP loading and initial citalopram concentration on the efficiency of the photodegradation process. Citalopram initial concentration was found to be the most significant parameter followed by irradiation time and pH, respectively. At optimum conditions, 88.43 ± 0.7% degradation of citalopram (25.00 µg/mL) was obtained in 1 h using UV light (1.01 mW/cm^2^). Citalopram kinetics of degradation followed pseudo-first order rate with *K*_obs_ and *t*_0.5_ of − 0.037 min^−1^ and 18.73 min, respectively. The optimized protocol was successfully applied for treatment of water samples collected during different cleaning validation cycles of citalopram production lines. The reusability of SnO_2_/GA NP was studied for 3 cycles without significant loss in activity. This approach would provide a green and economic alternative for pharmaceutical wastewater treatment of organic pollutants.

**Graphical abstract:**

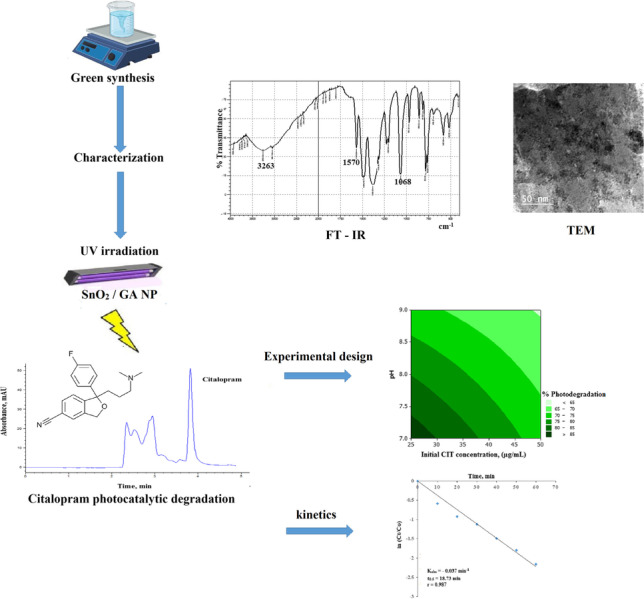

**Supplementary Information:**

The online version contains supplementary material available at 10.1007/s11356-022-22447-5.

## Introduction

**Fig. 1:**
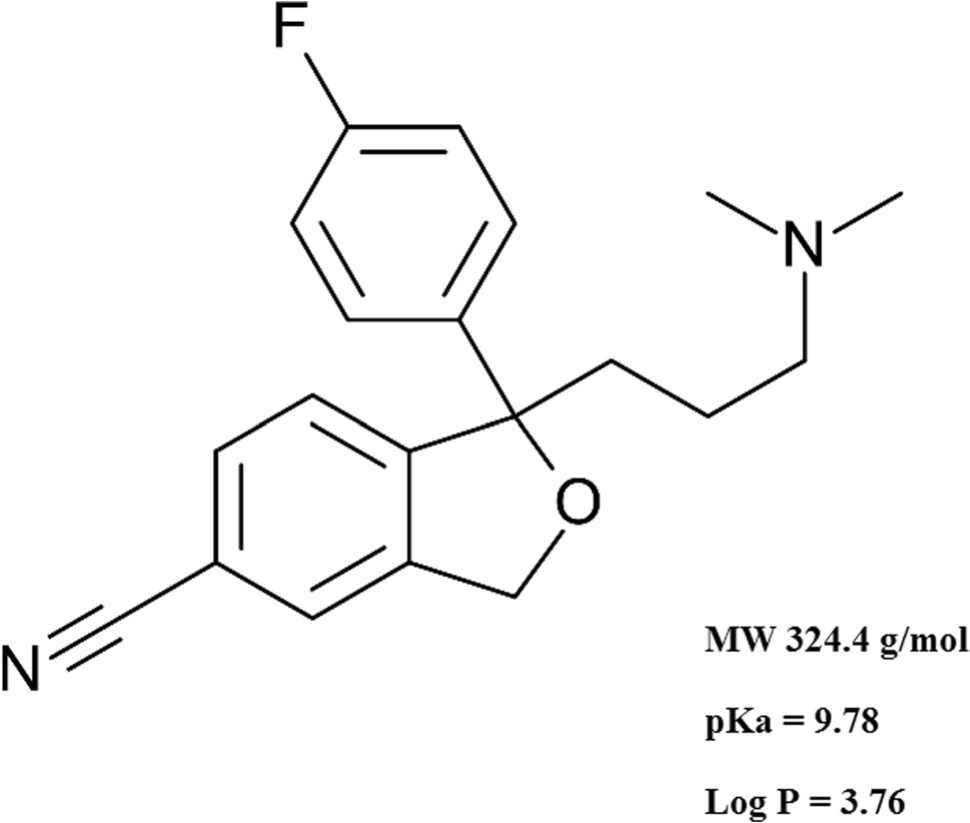
Chemical structure of Citalopram

Many pharmaceutical compounds have been detected in different water sources including sewage effluents, surface, ground and even drinking water (Gadipelly et al. 2014). This raised a concern about their potential risks to aquatic species, environment and human health (Carter et al. 2019). Citalopram (1-[3-(dimethylamino)propyl]-1-(4-fluorophenyl)-3H-2-benzofuran-5-carbonitrile) (CIT) is a selective serotonin reuptake inhibitor antidepressant drug (Fig. 1). It had been detected in various aquatic systems (Castillo-Zacarías et al. 2021; Giebułtowicz and Nałęcz-Jawecki 2014; Lajeunesse et al. 2012). Antidepressants have received high attention after COVID-19 pandemic which triggered the consumption of such class (Melchor-Martínez et al. 2021). Different analytical techniques were used to quantitatively determine CIT in aquatic samples. Among these methods are liquid chromatography (Sarıkaya et al. 2021), gas chromatography (Behpour et al. 2020), differential pulse voltammetry (Madej et al. 2019), tandem mass spectrometry (Evans et al. 2015) and capillary electrophoresis (Himmelsbach et al. 2006). In order to avoid undesired accumulation of CIT in aquatic environments, different treatment methods were employed such as adsorption (Ek et al. 2014; Guillossou et al. 2019; Sharifabadi et al. 2013), membrane bioreactor process (Arola et al. 2017) and gamma radiation (Bojanowska-Czajka et al. 2022). Photocatalysis is considered one of the most widely investigated advanced oxidation processes (AOPs) for removal of emerging contaminants. Photocatalytic degradation of CIT based on TiO_2_ nanoparticles (NP) had been reported (Jiménez-Holgado et al. 2021). However, it was limited by high recombination rate of photo-induced electronic–hole pairs produced upon ultraviolet (UV) irradiation and the relatively high cost of the photocatalyst (Han et al. 2009; Wu et al. 2015). SnO_2_ NP is one of the most promising photocatalysts. This could be attributed to its high oxidation potential, high photo-absorption ability, surface reactivity, chemical inertness, relative non-toxicity and long-term photochemical stability (Honarmand et al. 2019; Sun et al. 2022). Various methods had been reported for the synthesis of SnO_2_ NP such as hydrothermal (Akhir et al. 2019), sonochemical (Khan et al. 2017), microwave assisted methods (Sathishkumar and Geethalakshmi 2020). All the previously reported techniques had shown satisfactory synthesis outcome. However, those methods depended particularly on the use of surfactants and various toxic or hazardous chemicals. Green chemistry principles are of great interest to reduce the use of toxic methodologies for nanostructures development. Gallic acid (GA) (3,4,5 trihydroxybenzoic acid) is a naturally occurring polyphenolic antioxidant compound. It is one of the main constituents of tea leaves (Badhani et al. 2015) (Fig. S1). The use of GA for functionalization of various NP had been previously reported in a variety of applications (Lee et al. 2017; Nadim et al. 2015; Sarker et al. 2012). To the best of our knowledge, the use of SnO_2_ functionalized with GA as a photocatalyst in treatment of pharmaceutical wastewater has not been reported yet. Upon UV irradiation of GA, reactive oxygen species (ROS) can be generated (Benitez et al. 2005; Du et al. 2014; Luna et al. 2016; Wang et al. 2019). This would enhance the photocatalytic activity of SnO_2_.

In this study, GA was used to mediate the synthesis of SnO_2_ NP through a green approach, using a simple and economic method. GA was used not only as a reducing agent throughout the NP synthesis but also as a stabilizing agent through functionalization of SnO_2_ NP. The synthesized SnO_2_/GA NP were characterized and then used for the treatment of pharmaceutical wastewater containing CIT. A validated RP-HPLC assay was developed for monitoring CIT degradation throughout wastewater treatment. Optimization of the photocatalytic degradation process was carried out using full factorial experimental design. The kinetics of CIT degradation was studied. Application of the optimized protocol to incurred wastewater samples, collected during the pharmaceutical cleaning process of CIT production lines, was also investigated.

## Experimental

### Chemicals and samples

Stannous chloride dihydrate (SnCl_2_.2H_2_O) was purchased from Loba Chemie (Mumbai, India), whereas GA and titanium (IV) oxide NP (anatase, <25 nm) were purchased from Sigma-Aldrich (USA). CIT standard (purity 98.89% ± 0.53) as well as incurred water samples were kindly supplied by Delta Pharmaceutical Industries (Egypt). Two samples were obtained at two different stages of cleaning validation process: first wash of production lines using alkaline detergent and second wash using purified water. Double distilled water was used all through the synthesis of SnO_2_/GA NP. All other chemicals were of HPLC grade and obtained from Sigma-Aldrich (USA).

### Instruments

UV irradiation was performed using a 6 W-UV lamp with irradiation of 1.01 mW/cm^2^ (Vilber Lourmat, France) kept in an air-ventilated chamber. Agilent (1260 Infinity) HPLC system controlled with Chemstation software (Agilent Technologies, Germany) was used for chromatographic separations. HPLC analysis was performed using Xbridge Shield RP18 5 µm, 4.6 × 250 mm (Waters, Ireland). Fourier transform infrared (FTIR) spectroscopy was carried out using Shimadzu IRAffinity-1. TEM images of SnO_2_/GA NP were collected using a high-resolution transmission electron microscope (TEM 2100; JEOL, Japan). Zeta potential was measured using Zetasizer Nano ZS-ZEN 3600 (Malvern Instruments Ltd, UK). The statistical analysis and experimental design of the results were performed using Minitab, ver. 16.1.1 (Minitab Inc., USA). UV–Vis absorption spectrum was observed using Shimadzu 1650 spectrophotometer (Japan). X-ray diffraction (XRD) graph was recorded on a Bruker D8-Advance diffractometer (Bruker AXS Inc., USA). The surface area and pore size of NP were obtained from Brunauer–Emmett–Teller (BET) measurements using NOVA touch 4LX analyzer (Quantachrome, USA).

### Synthesis and characterization of SnO_2_/GA NP

A facile green chemical approach was used for the preparation of SnO_2_/GA NP by co-precipitation technique as previously reported but with slight modification (Tammina et al. 2019); 0.01 M GA aqueous solution was prepared and then added dropwise to 0.01 M SnCl_2_.2H_2_O aqueous solution. The two solutions were mixed and magnetically stirred at 500 rpm under a temperature of 90 °C for 6 h. A yellow-colored precipitate was produced then washed three times with water and centrifuged at 4000 rpm for 10 min to remove any unreacted substance. The precipitate was dried at 60 °C for 2 h and calcined at 300 °C for 2 h. Control SnO_2_ NP were prepared and recovered using the same procedure but with replacing GA with ammonia aqueous solution until pH had reached 9.0. Then, the mixture was stirred for 1 h at 90 °C. The synthesized SnO_2_/GA NP were inspected for their morphology using HR-TEM. One drop of the sample was put on a copper grid, air dried then examined at 200 kV. FT-IR was used to confirm the functionalization of SnO_2_ NP with GA. The sample was mixed with KBr to form flattened pellets. IR spectra were examined for the characteristic bands at 400–4000 cm^−1^. Average zeta potential was also determined using the Zetasizer at 25 °C for 120 s. Zeta potential measurements were carried out at pH range of 5.0, 7.0 and 9.0 in order to assess the colloidal stability of the NP. XRD graph of SnO_2_/GA NP was recorded on a Bruker D8-Advance diffractometer with backgroundless sample holders, and the X-ray generator was operated at 40 kV and 30 mA. Surface area measurements such as surface area (m^2^/g), total pore volume (cc/g), average pore radius (nm) and nitrogen adsorption–desorption isotherms of the SnO_2_/GA NP were also evaluated based on BET theory.

### Reversed-phase liquid chromatography

A previously reported HPLC assay (Skibiński and Misztal 2005) was used with some modifications so as to determine CIT in the presence of its photodegradation products. Briefly, optimal mobile phase condition was 50 mM dipotassium hydrogen phosphate buffer (pH 2.5 ± 0.1):acetonitrile (55:45%v/v). Isocratic elution was carried out at a flow rate of 1 mL/min, and CIT was detected at 239 nm. Calibration curve for CIT was constructed using standard series covering a concentration range of 0.50–25.00 µg/mL. Regression equation was obtained and used for calculation of residual CIT concentration all through the study. Validation was done according to ICH Guidelines: Q2(R1) (ICH Harmonized Tripartite Guidelines 2005). Validation parameters were determined: accuracy, precision, linearity and limit of detection. System suitability parameters were then calculated according to US Pharmacopoeia (The Unites States Pharmacopoeia and National Formulary 2011).

### Photocatalytic degradation study

#### Preliminary studies

Standard CIT samples were prepared (50.00 µg/mL) in phosphate buffer pH 5.0, 7.0 and 9.0 and were left in dark at room temperature for 2 h to assess CIT hydrolytic stability. Then, two CIT samples (50.00 µg/mL) were prepared in phosphate buffer pH 7.0 and exposed to UV irradiation (254 nm, 1.01 mW/cm^2^) for 1 h in the absence and presence of SnO_2_/GA NP (0.50 mg/mL). For comparative purposes, same experimental conditions were applied for two CIT control samples using (i) commercially available TiO_2_ NP and (ii) bare SnO_2_ NP. To assess the effect of pH on the activity of the photocatalyst, CIT samples (50.00 µg/mL) were prepared in phosphate buffer pH 5.0 and 9.0 then exposed to UV irradiation for 1 h. Finally, all samples had been analyzed using the described HPLC assay.

#### Adsorption isotherm

Adsorption equilibrium experiments were performed in dark conditions using 25-mL amber vials. Aliquots of 10 mL of various initial CIT concentrations (10.00–50.00 µg/mL, pH 7.0) were kept in contact with fixed concentration of SnO_2_/GA NP (0.5 mg/mL) under constant magnetic stirring for 30 min. Then, the solutions were filtered and analyzed by HPLC.

#### Experimental design

The effects of irradiation time, pH, SnO_2_/GA NP loading and initial CIT concentration were studied. Two levels were randomly assigned for each of the four factors (2^4^) either low (− 1) or high (+ 1) as presented in Table 1. Sixteen sets of experimental conditions (two-level full factorial design 2^4^) were conducted as illustrated in Table 2. All experiments were performed at room temperature, in air ventilated cabinet while continuously magnetically stirred. Before exposure to UV irradiation, CIT and NP mixture was stirred in the dark for 30 min to achieve adsorption equilibrium. All through the study, UV irradiation of 25 mL of buffered CIT solution and SnO_2_/GA NP was carried out as indicated in each experiment in Table 2. At the end of the incubation period, samples were completed to volume (25 mL) and filtered through a syringe filter (0.2 µm). Samples were then analyzed using the developed RP-HPLC assay.

**Table 1:** Actual factors and the levels used in two-level full factorial design experiment

Factor name	Factor code	Low level (− 1)	High level (+ 1)
Time (h)	A	1	2
SnO_2_/GA loading (mg/mL)	B	0.5	1
pH	C	7	9
Initial CIT concentration (µg/mL)	D	25	50

**Table 2:** Design matrix for 2^4^ full factorial experimental design constructed for photocatalytic degradation of CIT and results obtained through RP-HPLC assay

Run no	Factor code				RP-HPLC	
	A	B	C	D	CIT concentration (µg/mL)	% Photodegradation
1	+ 1	− 1	− 1	− 1	2.51	89.97
2	+ 1	+ 1	− 1	+ 1	8.85	82.30
3	− 1	+ 1	+ 1	+ 1	16.08	67.85
4	+ 1	− 1	+ 1	− 1	3.37	86.52
5	− 1	+ 1	+ 1	− 1	5.08	79.68
6	− 1	− 1	− 1	+ 1	13.80	72.41
7	+ 1	+ 1	+ 1	− 1	3.26	86.97
8	− 1	− 1	− 1	− 1	2.89	88.43
9	+ 1	− 1	+ 1	+ 1	12.64	74.73
10	− 1	+ 1	− 1	+ 1	15.31	69.38
11	+ 1	+ 1	− 1	− 1	2.10	91.61
12	− 1	+ 1	− 1	− 1	4.41	82.35
13	− 1	− 1	+ 1	+ 1	17.46	65.09
14	+ 1	+ 1	+ 1	+ 1	11.11	77.79
15	− 1	− 1	+ 1	− 1	7.15	71.42
16	+ 1	− 1	− 1	+ 1	11.75	76.50

### Kinetics of CIT photocatalytic degradation

Kinetics of CIT photocatalytic degradation reaction was studied over 1 h at 10-min intervals at the optimum set of conditions: CIT (25.00 µg/mL, pH 7.0 ± 0.1) in the presence of SnO_2_/GA NP (0.50 mg/mL). RP-HPLC assay was used to monitor CIT concentration gradual decrease over time.

### Application to incurred samples

After one production batch of CIT tablets, cleaning of production lines was conducted according to the manufacturer’s protocols. Two washing cycles were implemented for cleaning validation. A commercially available alkaline wash solution (Alcojet ®) was used in the first cycle, followed by purified water in the second cycle. Pooled samples were collected during each washing cycle and stored at − 20 °C. Upon analysis, the pH of the samples was adjusted to 7.0, and CIT concentration was then determined. Subsequently, the samples (25 mL each) were subjected to UV irradiation in the presence of SnO_2_/GA NP (0.50 mg/mL) for 1 h. After the irradiation period, CIT concentration and the percent of degradation were determined.

## Results and discussion

### Synthesis and characterization of NP

An ecofriendly SnO_2_/GA NP was synthesized using a facile co-precipitation method. GA was employed as a reducing agent for SnO_2_. This eliminated the use of any hazardous reducing agents and reduced the generation of toxic byproducts. GA was also employed as a stabilizing agent for SnO_2_. The functionalized NP with GA exhibited high photocatalytic efficiency owing to the ability of GA to generate ROS such as hydrogen peroxide and hydroxyl radicals upon UV irradiation (Benitez et al. 2005; Du et al. 2014; Luna et al. 2016; Wang et al. 2019). Thus, GA enhanced the stability as well as the catalytic activity of SnO_2_ NP. It should be noted that allowing the reaction mixture for 4 h had only resulted in reduction of SnCl_2_ (Tammina et al. 2019). Increasing the synthesis time up to 6 h had resulted in surface functionalizing of SnO_2_ NP with GA. This came into agreement with previous literature of functionalizing metal oxides NP with carboxylic acid moieties (Lee et al. 2017; Sarker et al. 2012). Further increase of the reaction time to 8 h gave the same yield and photocatalytic activity of NP. Therefore, 6-h synthesis was chosen as optimum.

**Fig. 2:**
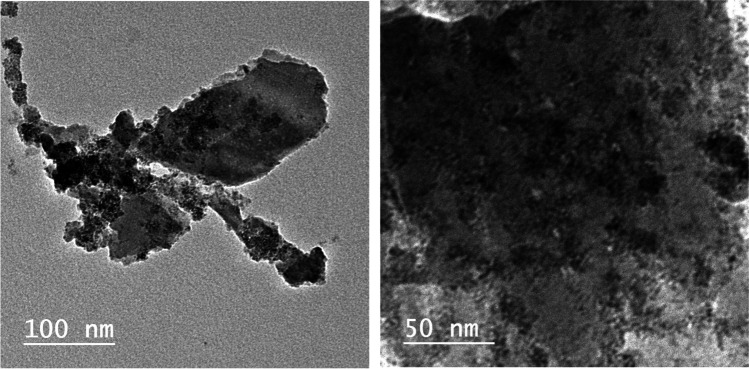
Transmission electron micrograph of SnO_2_/GA NP

**Fig. 3:**
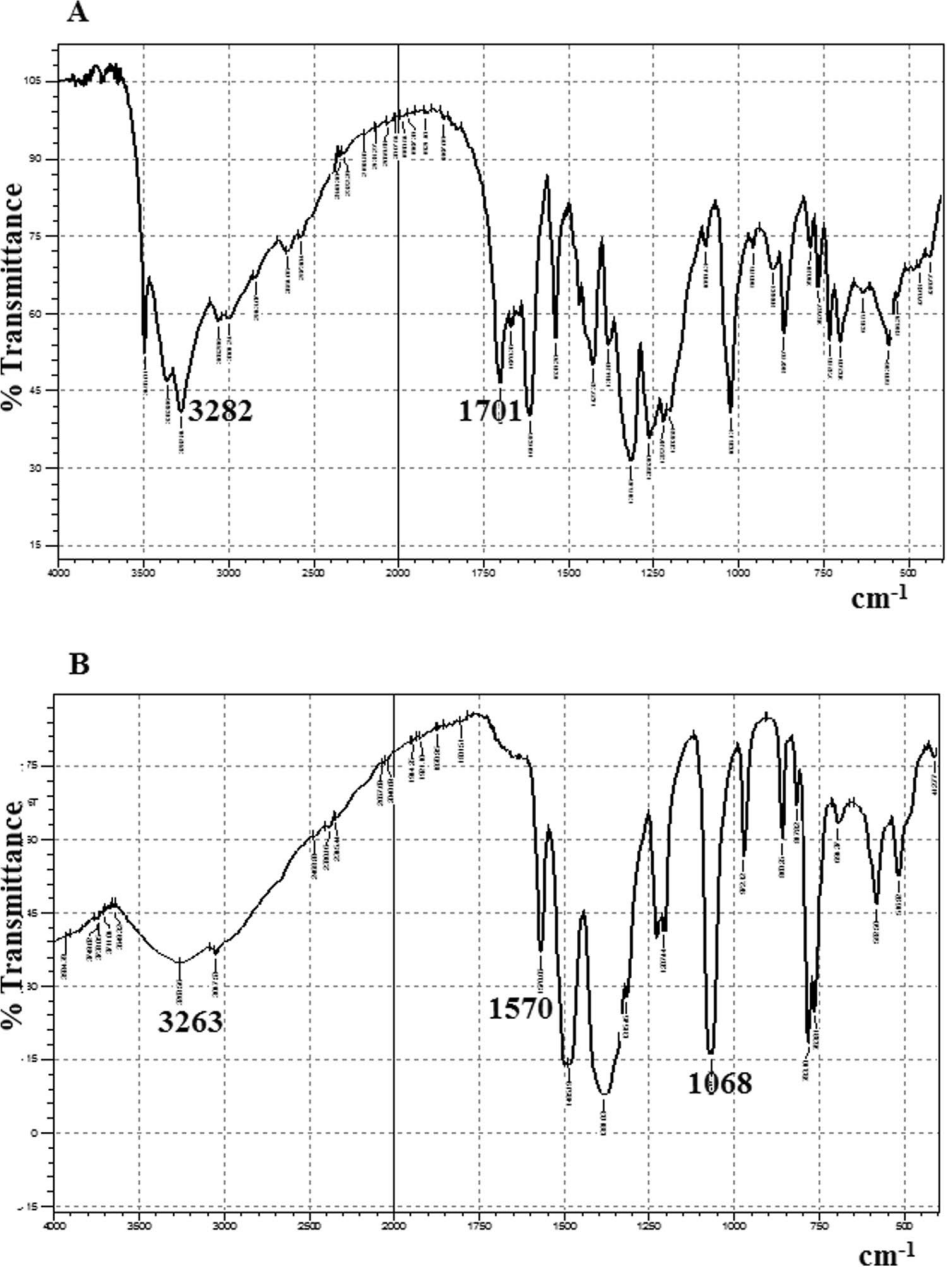
FT-IR spectra of (**A**) pure GA and (**B**) SnO_2_/GA NP

**Fig. 4:**
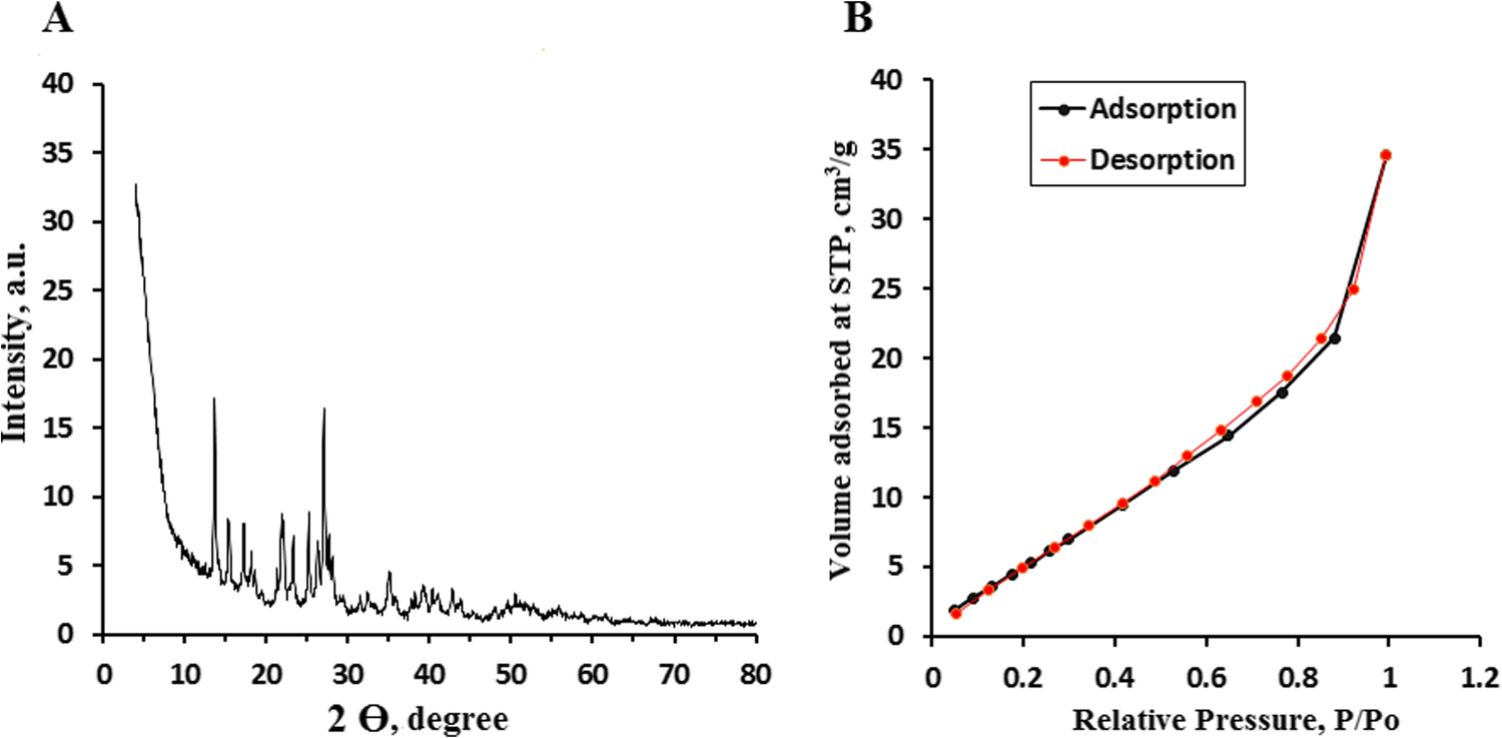
(**A**) XRD graph of SnO_2_/GA NP. (**B**) Nitrogen adsorption–desorption isotherms surface area of SnO_2_/GA NP

The morphology and detailed crystal structure of SnO_2_/GA NP were investigated using HR-TEM (Fig. 2). Results showed the formation of spherical SnO_2_ NP coated with GA with a mean hydrodynamic diameter of 10 ± 3.85 nm. FTIR spectrum of pure GA was compared to that of SnO_2_/GA NP to confirm binding between GA and SnO_2_ (Fig. 3). Pure GA spectrum showed a broad peak at 3282 cm^−1^ due to carboxylic and phenolic hydroxyl groups (–OH) stretching. A sharp peak at 1701 cm^−1^ was observed indicating carbonyl (C = O) stretching of COOH group. Compared to SnO_2_/GA NP spectrum, the broad peak of (–OH) stretching was observed at 3263 cm^−1^ but with remarkable decrease in intensity. This indicated the interaction of hydroxyls groups in the functionalization of SnO_2_. A peak at 1570 cm^−1^ was also observed due to C = C aromatic stretching. Peaks were also observed at 1068 and 1485 cm^−1^ because of C–O and C–C stretching, respectively. These results indicated the formation of SnO_2_/GA NP (Sarker et al. 2012). Zeta potential measurement was also used to investigate the surface charge and the colloidal stability of SnO_2_/GA NP. Measurements were carried out at acidic (5.0), neutral (7.0) and alkaline pH (9.0). Optimum potential was achieved at pH 7.0 (− 30.8 ± 1.5 mv) which was sufficient to avoid aggregation. As the pH increased to 9.0, the value of zeta potential decreased to − 18.5 ± 3 mv, while pH 5.0 showed the least colloidal stability with zeta potential of − 14.4 ± 0.9 mv. The high negative charge at neutral pH was sufficient enough to form an intra molecular repulsive barrier. The absorption spectra of SnO_2_/GA NP showed an intense absorption in the range of 220–300 nm with a peak at 260 nm corresponding to GA UV absorption (Fig. S2). The crystalline structure of the synthesized SnO_2_/GA NP was analyzed by XRD (Fig. 4a). Data showed diffraction peaks at 2Ɵ, and the corresponding plane coordinates were 25.19° (112), 26.95° (211), 29.72° (202), 35.56° (310), 37.14° (311), 39.49° (004), 46.90° (313). This confirmed that the main composition of the nanoparticles was SnO_2_ (Ma et al. 2020). Also, some peaks for GA had appeared at 2Ɵ = 15.35°, 17.23° and 21.85°. This reflected the successful modification of the surface of SnO_2_ NP and confirmed the functionalization of SnO_2_ NP with GA (Hu et al. 2013; Patil and Killedar 2021). BET analysis revealed that the surface area and pore radius were 28.35 m^2^/g and 1.92 nm, respectively with total pore volume of 0.72 cc/g. The high surface area of NP obtained had allowed better contact for CIT and thus high photocatalytic properties. Nitrogen adsorption–desorption isotherm showed type IV isotherm with H3 hysteresis loop which is characteristic for mesoporous materials (Fig. 4b).

### Reversed-phase liquid chromatography

A CIT photodegraded sample was prepared and used for optimization of the RP-HPLC assay. Good resolution was obtained over 5 min using the assay conditions described above. An equivalent CIT sample was prepared and had not been subjected to UV irradiation as a control sample to verify the identity of CIT and calculate the percentage degradation as well. System suitability parameters were computed according to the US Pharmacopoeia (Table 3). Validation parameters and regression equation were also summarized in Table 3.

**Table 3:** Summary of system suitability and validation parameters for the developed RP-HPLC assay

System suitability parameters ^a^	
Retention time (min)	3.84
Asymmetric factor (*T*)	0.72
Selectivity (*α*)	1.30
Resolution (*Rs*)	4.93
Capacity factor (*k*´)	1.13
Number of theoretical plates (*N*)	7572
Height equivalent to a theoretical plate (HETP)	0.03
Validation parameters	
Accuracy (mean ± SD)^b^	100.85 ± 1.01
Precision (%RSD)	
Repeatability ^c^	0.525
Intermediate precision ^d^	1.747
Linearity	
Regression equation	y = 75.667x + 84.865
Correlation coefficient (*r*)	0.9998
Range (µg/mL)	0.50—25.00
LOD (µg/mL) ^e^	0.01
LOQ (µg/mL) ^f^	0.24
Robustness (mean ± SD) ^g^	
Asymmetric factor (*T*)	0.55 ± 0.01
Capacity factor (*k*´)	1.15 ± 0.17

^a^Reference values for HPLC parameters: *k*` ≥ 1, *α* ≥ 1, *Rs* ≥ 1.5, *T* ≤ 2 and *N* ≥ 2000

^b^Average percentage recovery of nine determinations over three concentration levels

^c^The intraday, average of nine determinations over three concentration levels repeated three times within the same day

^d^The interday, average of nine determinations over three concentration levels repeated three times over three different days

^e^LOD determined via calculations, 3.3 (SD of the response/slope)

^f^LOD determined via calculations, 10 (SD of the response/slope)

^g^Average of nine determinations over three concentration levels

### Photocatalytic degradation study

#### Preliminary studies

**Fig. 5:**
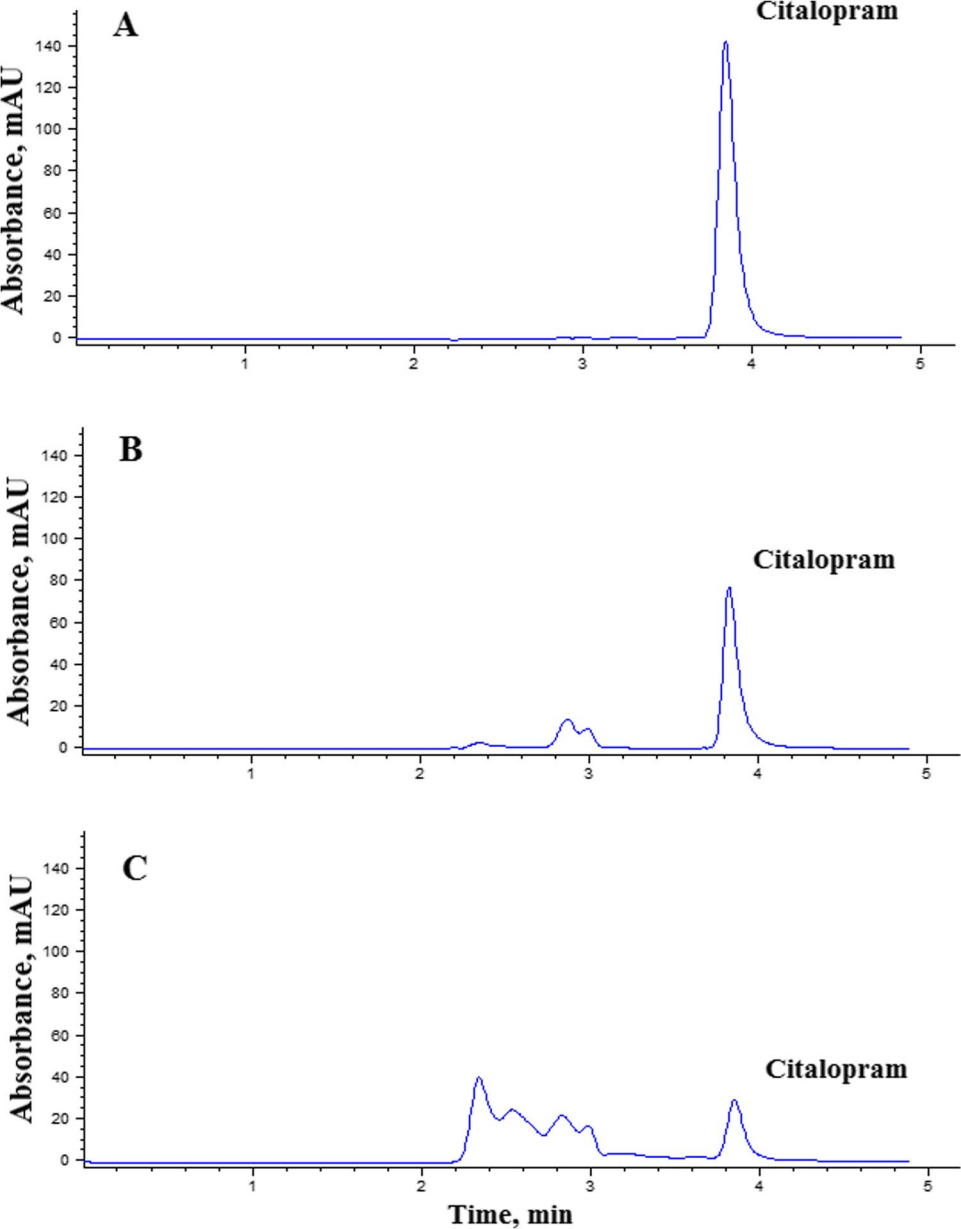
HPLC chromatogram of (**A**) CIT sample (50.00 μg/mL) not subjected to UV irradiation. (**B**) CIT degradation upon exposure to UV light intensity (1.01 mW/cm^2^) at pH 7.0 for 1 h in the absence of SnO_2_/GA NP. (**C**) CIT photocatalytic degradation upon exposure to UV light intensity (1.01 mW/cm^2^) at pH 7.0 for 1 h in the presence of 0.50 mg/mL SnO_2_/GA NP

Initially, CIT hydrolytic stability at pH 5.0, 7.0 and 9.0 was confirmed at room temperature over 2 h (data not shown). Results were in agreement to the previously reported data showing the relative stability of CIT (Kwon and Armbrust 2005). Then, CIT samples (50 µg/mL) were subjected to UV irradiation at pH 7.0 for 1 h in the absence and presence of NP. In the presence of UV light only, 29% degradation was obtained while the addition of SnO_2_/GA NP had increased percentage degradation to 71% (Fig. 5). In the presence of a control sample (50.00 µg/mL, pH 7.0) containing TiO_2_ NP as photocatalyst, only 61% degradation was noted, while in the presence of bare SnO_2_ NP, 49% degradation was obtained. These results showed the possible promising effect of the synthesized SnO_2_/GA NP. Preliminary screening of the effect of pH was carried out in the presence of NP. A relatively low (47%) degradation was obtained at pH 5.0 owing to the colloidal stability of the NP as explained earlier, while the % degradation had increased to 71% and 65% at pH 7.0 and 9.0 respectively. Therefore, pH 7.0 and 9.0 were further studied.

#### Adsorption isotherm

**Fig. 6:**
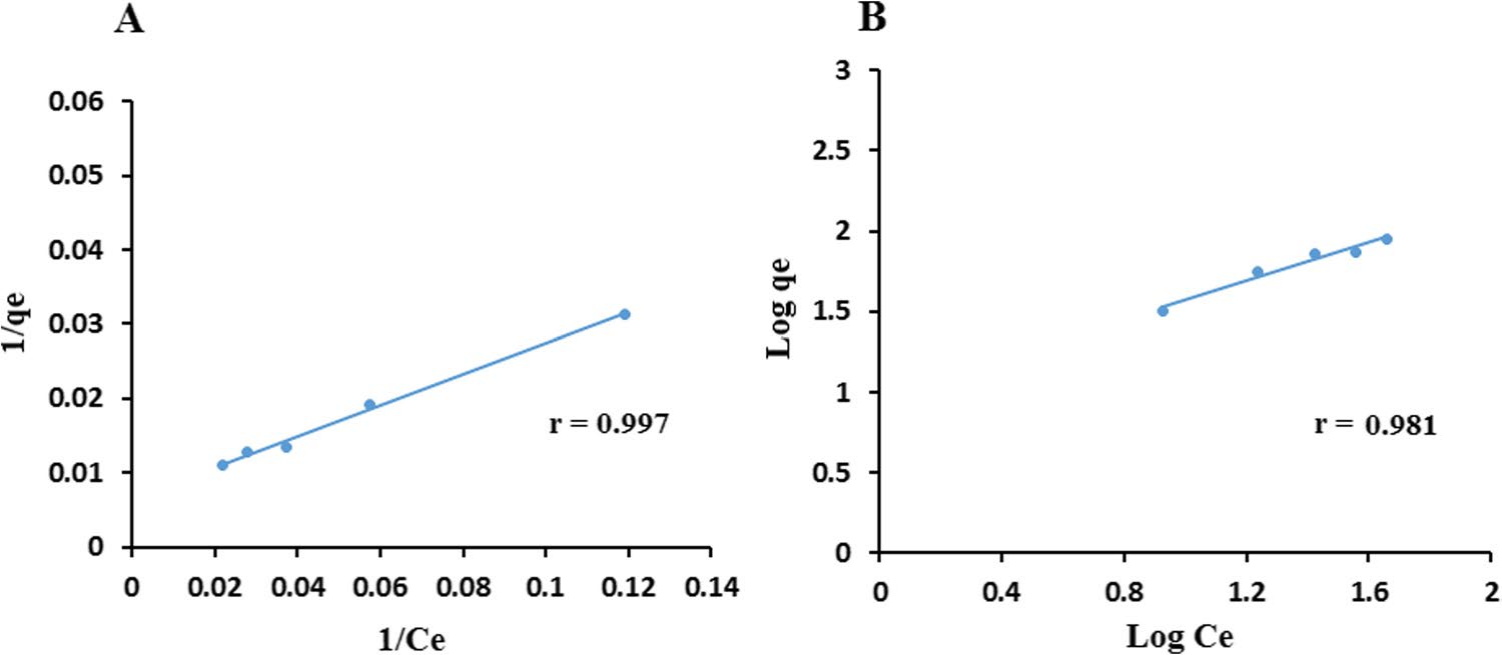
Adsorption isotherm of CIT on SnO_2_/GA NP using (**A**) Langmuir model and (**B**) Freundlich model

Initial experiments performed for 1 h under constant magnetic stirring indicated that the adsorption equilibrium was achieved after 25 min. No further adsorption was observed after 30 min. Adsorption in dark conditions had contributed to 16.42% removal of CIT. To investigate the interaction of CIT molecules and the adsorbent surface of NP, two well-known models, Langmuir and Freundlich isotherms, were studied to describe CIT adsorption equilibrium. The Langmuir isotherm is valid for monolayer adsorption onto a surface with a finite number of identical sites (Bouafıa-Cherguı et al. 2016; Meroufel et al. 2013). It is given as the following Eq. $1/Qe=1/Qmax+1/\left(Qmax\times {K}_{L}\right)\times 1/Ce$. *Qe* is the adsorbed quantity of CIT (mg/g), *Ce* is CIT concentration (mg/L) at the adsorption equilibrium, *K*_*L*_ is the Langmuir adsorption constant in the dark (L/mg) and *Q*max is the maximum adsorbed quantity of CIT (mg/g). Upon plotting 1/*Qe* against 1/*Ce* (Fig. 6a), a straight line was obtained, and the Langmuir isotherm provided a good fit of the data. The Freundlich isotherm equation is log *Qe* = log *K*_*F*_ + 1/*n* log *Ce*. *K*_*F*_ and *n* are the constants of adsorption density and adsorption intensity, respectively (Fig. 6b). The Freundlich isotherm gives no information on the monolayer adsorption density. Langmuir isotherm model was found to be slightly better for describing the adsorption equilibrium than Freundlich model (Table 4).

**Table 4:** Adsorption isotherm parameters of CIT on SnO_2_/GA NP

Langmuir isotherm constants	Freundlich isotherm constants
*Q* max = 151.51 mg/g	*K*_*F*_ = 9.749 L/mg
*K*_*L*_ = 0.033 L/mg	*n* = 1.704
*r* = 0.997	*r* = 0.981

#### Experimental design

Further investigation was conducted in order to optimize the effects of (A) irradiation time, (B) SnO_2_/GA NP loading, (C) pH and (D) initial CIT concentration on the efficiency of CIT photodegradation. A relatively high concentration of CIT up to 50.00 µg/mL was used to cover the expected range in pharmaceutical wastewater. NP loading of 0.50 mg/mL was chosen as it is a commonly starting point in different photocatalytic procedures (Lu et al. 2019a; Mugunthan et al. 2019; Štrbac et al. 2018). Full factorial design was used to assess the relative significance of the mentioned factors, their interactions as well as the optimal set of experimental conditions for CIT photocatalytic degradation (Table 1). Samples were then analyzed in duplicate using the developed RP-HPLC assay, and the percentage degradation was shown in Table 2.

**Fig. 7:**
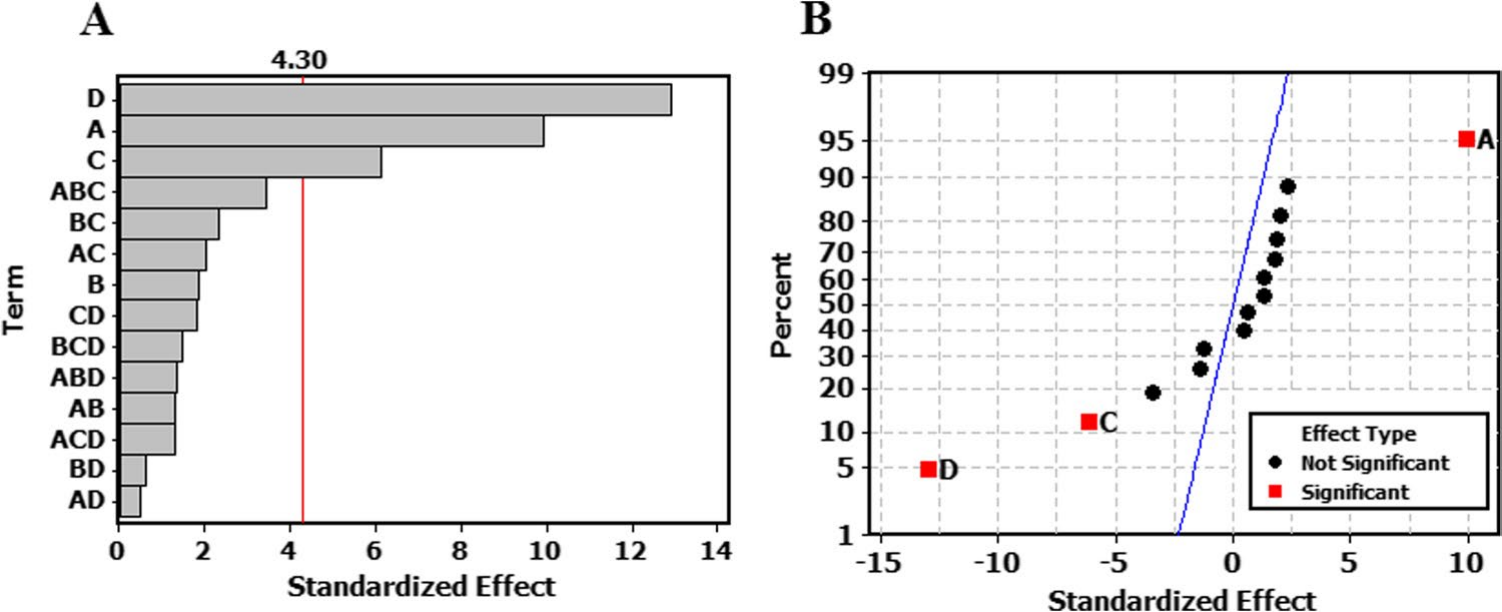
(**A**) Pareto chart of the standardized effects of single and interaction factors on CIT photodegradation. (**B**) Normal plot of the standardized effects of single and interaction factors on CIT photodegradation

**Fig. 8:**
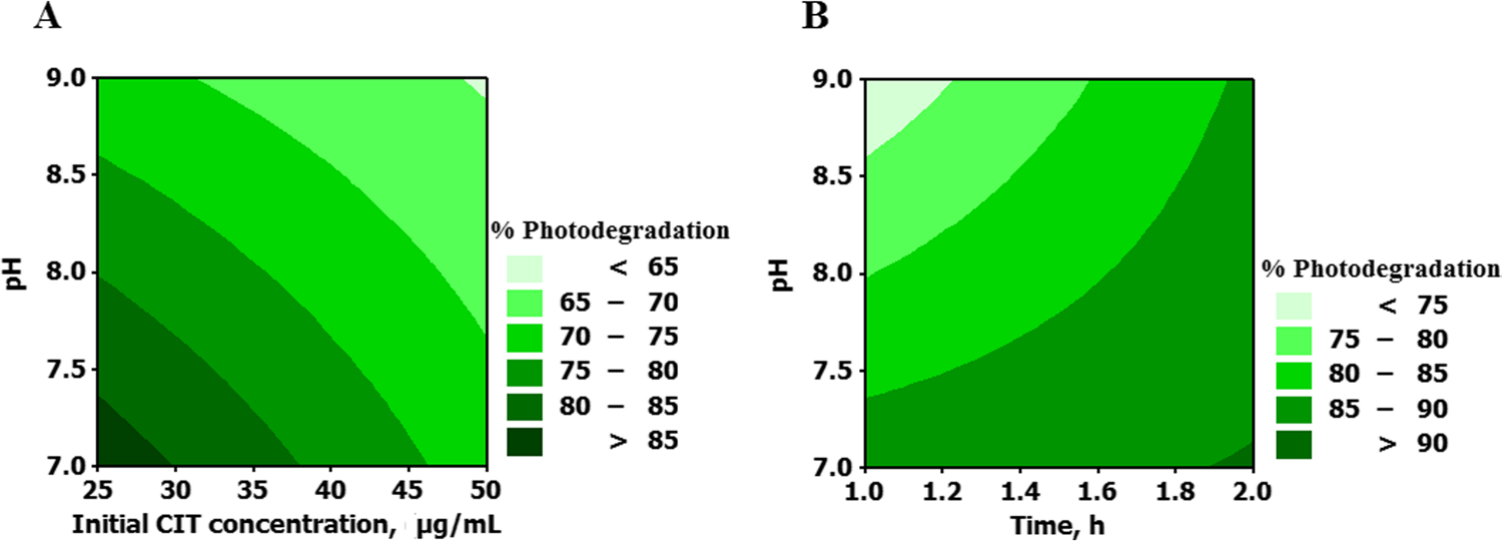
Contour plot for pH versus (**A**) CIT initial concentration (hold values: UV irradiation time 1 h, SnO_2_/GA NP loading 0.50 mg/mL). (**B**) UV irradiation time (hold values: initial concentration 25.00 μg/mL, SnO_2_/GA NP loading 0.50 mg/mL)

Analysis of full factorial design results was done at 95% confidence level (*P* 0.05) using the percentage of degradation as the response factor. The relative magnitude of the studied factors and their interactions was visualized using pareto diagram, while the direction of effects was illustrated by the normal plot of the standardized effects. In this study, irradiation time (A) was found to have a significant impact with a positive effect indicating an increase in photocatalysis at high levels, while pH (C) and initial CIT concentration (D) have a significant impact with a negative effect revealing a decrease in response at high levels of these variables (Fig. 7). It should be noted that the interactions ABC (irradiation time-catalyst-pH) showed no significant effect but with *P* value of 0.076 as shown in Fig. 7 and Table S1. pH 7.0 showed superior catalytic efficiency compared to pH 9.0. At neutral conditions, CIT is fully ionized with positive charge (CIT pKa = 9.78) enabling facile attraction to the negatively charged catalyst, while at pH 9.0, the drug is partially ionized, and the NP is more aggregated. In addition, increasing SnO_2_/GA NP loading to 1.0 mg/mL was found to be non-significant. This could suggest a plateau effect for the catalyst over a range of 0.50–1.00 mg/mL. It could be concluded that the longer the irradiation time at neutral conditions, the higher percentage of photodegradation observed (Fig. S3). The same degradation efficiency (~ 82%) was obtained for CIT 25.00 and 50.00 µg/mL but when irradiated for 1 and 2 h respectively. This indicated that treating samples containing low initial concentrations of CIT are required to improve the economics and efficiency and of the treatment process. This was illustrated in the surface plot (Fig. S3). Contour diagram was also constructed to predict the optimum experimental region for CIT photodegradation (Fig. 8). Maximum photodegradation can be achieved at pH 7.0 using lower initial CIT concentration (25.00–30.00 μg/mL) (Fig. 8a). Similarly, Fig. 8b showed that maximum photodegradation can be obtained after 2 h at the same pH.

Maximum photodegradation (91%) was obtained at pH 7.0 in the presence of 25.00 µg/mL CIT and 1.00 mg/mL NP in 2 h. However, 88% degradation was obtained in 1 h at the same conditions except using less NP loading 0.50 mg/mL. Therefore, increasing the irradiation time by another 1 h has minimum effect ( ~ 3%) on the photocatalytic efficiency. In order to improve the economics of CIT photodegradation, the 1-h treatment protocol using 0.50 mg/mL NP was chosen as optimum set and was used for further investigations (Table 2).

The regression equation summarizing the experimental design is given as follows:

$$Y=354.488-98.9566A-223.433B-35.0314C-2.95526D+86.9269AB+14.1748AC+0.478808AD+29.6711BC+1.15004BD+0.349987CD-12.0297ABC+0.368721ABD-0.0901978ACD-0.202070BCD$$

where *Y* = % degradation, *A* = irradiation time, *B* = SnO_2_/GA NP loading, *C* = pH and *D* = initial CIT concentration.

Analysis of variance (ANOVA) was carried out, and the results were calculated in Table S1. A lack of fit value of 0.498 was observed indicating its non-significance and an acceptable predictability of the studied model.

Upon treatment of CIT samples (25.00 µg/mL, pH 7.0) with a commercially available TiO_2_ NP (0.50 mg/mL) for 1 h, 76% degradation was obtained. It should be noted that a previously reported literature had used a commercially available TiO_2_ NP for treatment of CIT (20.00 µg/mL) in water samples. Although full degradation was reported in 30 min, a high-intensity UV lamp was used (9 mW/cm^2^) compared to the low energy lamp used in this study (1 mW/cm^2^). This indicated that the synthesized SnO_2_/GA NP could spare the need of high-watt UV lamps. In addition, different forms of SnO_2_ NP had been used as photocatalysts with acceptable degradation efficiency. However, either lengthy procedure, hazardous chemicals, high energy UV light sources were used or small molecular weight organic molecules/ dyes were used as the studied model (Table 5). Although satisfactory results were obtained in our study under UV light, the synthesized SnO_2_/GA NP can be further modified in the future to work under visible range for an extra economical advantage.

**Table 5:** Summary of reported methods for SnO_2_ photocatalysis

SnO_2_ NP	Studied model	Degradation efficiency	Comment	Ref
SnO_2_/GA	Citalopram	88% in 60 min	- Green simple synthesis - Low-watt UV consumption (1.01 mW/cm^2^)	This work
SnO_2_ NP (using chitosan)	Eriochrome Black T	77% in 270 min	- High-watt UV lamp was used (500 W)	(Najjar et al. 2021)
SnO_2_ quantum dots encapsulated carbon nanoflakes	Bisphenol A	98% in 60 min	- Green multi-step synthesis - Synergistic adsorption and photodegradation process - Relatively high-watt UV consumption (3.47 mW/cm^2^)	(Mohanta and Ahmaruzzaman 2020)
AgBr/SnO_2_ nanocomposite	Rhodamine B and caffeic acid	95% in 45 min	- High cost of Ag used - High-watt UV consumption (90,000 mW/cm^2^)	(Puga et al. 2021)
Flower-like SnO_2_ nanocomposites	Methyl orange and rhodamine B	95% in 90 min	- Hazardous chemicals were used - High-watt UV lamp was used (250 W)	(Lu et al. 2019b)
Strontium-doped SnO_2_ NP	- Methylene blue - Dinoseb	94% in 60 min 82% in 120 min	- Hazardous chemicals were used - High-watt UV lamp was used (125 W)	(Ahmed et al. 2019)
Quasi-monodispersed SnO_2_ microspheres	Rhodamine B	98% in 210 min	- Hazardous chemicals were used - High-watt UV lamp was used (300 W)	(Zhu et al. 2014)

### Kinetics of CIT photocatalytic degradation

**Fig. 9:**
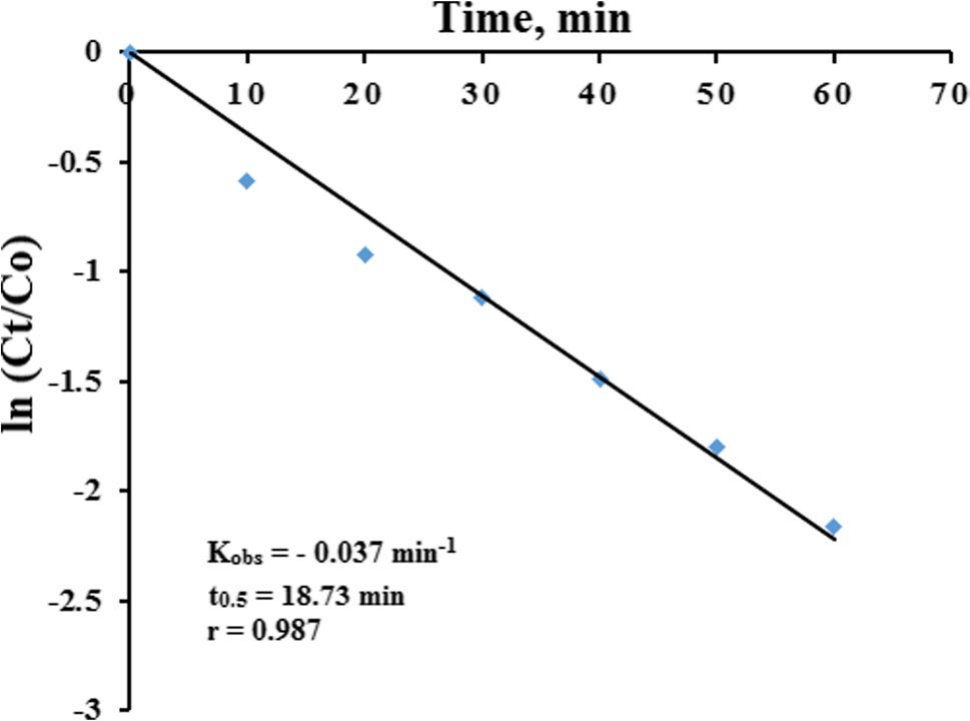
Kinetics profile of CIT photodegradation with initial CIT concentration 25.00 µg/mL at pH 7.0 with 0.50 mg/mL SnO_2_/GA NP

The kinetics of CIT photocatalytic degradation was studied at optimum set of experimental conditions (25.00 µg/mL CIT, pH 7.0, 0.50 mg/mL NP, 1 h). Upon plotting ln (*Ct*/*Co*) against time, a straight line was obtained which indicated a pseudo-first order kinetics. By applying Langmuir–Hinshelwood model (Gaya and Abdullah 2008), a relatively fast kinetics profile was obtained with *K*_obs_ and *t*_0.5_ of − 0.037 min^−1^ and 18.73 min, respectively (Fig. 9).

### Reusability of SnO_2_/GA NP

The capability to regenerate SnO_2_/GA NP was investigated. SnO_2_/GA (0.50 mg/mL) was added to standard CIT buffered solution (25.00 µg/mL in phosphate buffer pH 7) and UV irradiated for 1 h. Then, the sample was centrifuged, washed well with double distilled water and left to dry in air for subsequent use. The recovered SnO_2_/GA NP were reused in second and third cycles for photocatalytic degradation of CIT under the same experimental conditions. Minimum decrease (~ 9%) in the photocatalytic activity was observed after three cycles (Fig. S4). Therefore, the reusability of SnO_2_/GA NP did not significantly affect its catalytic activity offering an economical advantage.

### Application to incurred samples

Incurred samples were collected during first wash cycle of the production lines, and CIT initial concentration was 35.22 ± 0.17 μg/mL, whereas CIT initial concentration in samples collected during second wash was found to be 1.20 ± 0.01 μg/mL. The developed photocatalytic degradation was used as a treatment protocol and investigated using first wash samples. The percentage of degradation was found to be 81.44 ± 0.61% and 82.35% ± 0.42% for the incurred and control samples under the optimum conditions. The absence of significant difference revealed lack of matrix interference. Upon application of the treatment protocol to second wash sample, CIT peak was not detected owing to its relatively low concentration (less than the assay LOD). These results showed the efficiency of SnO_2_/GA NP as a photocatalyst in treatment of wastewater collected during CIT cleaning validation.

## Conclusion

SnO_2_/GA NP were employed as a novel and eco-friendly photocatalyst for treatment of pharmaceutical wastewater containing CIT. Synthesis was performed using a green, simple and low-cost chemical approach using GA. No hazardous chemicals were required during NP preparation. A percentage degradation of 88% was obtained in 1 h using a low-energy UV light source. SnO_2_/GA NP had the advantage of being effective, recyclable and economic. Analysis of full factorial design had revealed that the most prompting factors affecting the photodegradation process were initial CIT concentration, UV irradiation time and pH. The treatment protocol was successfully applied for samples collected during the cleaning validation process of CIT production lines. This protocol would offer a cost-effective and energy efficient platform for photocatalytic treatment of wastewater.

## Supplementary Information

Below is the link to the electronic supplementary material.

Supplementary file1 (DOCX 22 KB)

ESM 1 (PNG 11 kb)

High resolution image (TIF 28 kb)

ESM 2 (PNG 8 kb)

High resolution image (TIF 27 kb)

ESM 3 (PNG 41 kb)

High resolution image (TIF 59 kb)

ESM 4 (PNG 9 kb)

High resolution image (TIF 29 kb)

## Data Availability

The data that support this study are available in the article and accompanying online supplementary material.
