# Supplementary material for: Functionalized SnO2 nanoparticles with gallic acid via green chemical approach for enhanced photocatalytic degradation of citalopram: synthesis, characterization and application to pharmaceutical wastewater treatment
Source: Environ Sci Pollut Res Int. 2022 Aug 15;30(2):4346–58. doi: 10.1007/s11356-022-22447-5 (PMC9376129; doi:10.1007/s11356-022-22447-5)
Supplement: Supplementary file 1 — Supplementary file1 (DOCX 22 KB) [file 11356_2022_22447_MOESM1_ESM.docx]

**Supplementary materials**

**Functionalized SnO_2_ Nanoparticles with Gallic acid via Green Chemical Approach for Enhanced Photocatalytic Degradation of Citalopram: synthesis, characterization and application to pharmaceutical wastewater treatment**

Veronia S. Nazim^a^, Ghada M. El-Sayed^a^, Sawsan M. Amer^a^, Ahmed H. Nadim^a*^

^a^  *Analytical Chemistry Department, Faculty of Pharmacy, Cairo University,, Egypt*.

***Correspondence:**

Ahmed H. Nadim,

Faculty of Pharmacy - Cairo University

Kasr El-Aini st, Cairo, EGYPT

ahmed.nagib@pharma.cu.edu.eg

**Figure captions**

Fig. S1: Chemical structure of gallic acid.

Fig. S2: UV-Vis absorption spectrum of SnO_2_/GA NP.

Fig. S3: 3D surface plot of CIT % photodegradation versus (A) initial CIT concentration – time, (B) time – pH and (C) SnO_2_/GA NP loading – irradiation time.

Fig. S4: Photocatalytic degradation percentage of CIT after three successive runs.

**Tables**

Table S1: Analysis of variance for photocatalytic degradation of CIT.

| **Source of variation** | **Degree of freedom** | **Sum of squres** | **Mean of squares** | **F value** | **P value *** |
| --- | --- | --- | --- | --- | --- |
| **Main effects** | 4 | 946.556 | 236.639 | 76.82 | 0.013 |
| **A** | 1 | 304.496 | 304.496 | 98.85 | 1.010 |
| **B** | 1 | 10.345 | 10.345 | 3.36 | 0.208 |
| **C** | 1 | 115.053 | 115.053 | 37.35 | 0.026 |
| **D** | 1 | 516.661 | 516.661 | 167.73 | 0.006 |
| **2 way interactions** | 6 | 45.877 | 7.646 | 2.48 | 0.315 |
| **AB** | 1 | 5.100 | 5.100 | 1.66 | 0.327 |
| **AC** | 1 | 12.533 | 12.533 | 4.07 | 0.181 |
| **AD** | 1 | 0.713 | 0.713 | 0.23 | 0.678 |
| **BC** | 1 | 16.394 | 16.394 | 5.32 | 0.147 |
| **BD** | 1 | 1.171 | 1.171 | 0.38 | 0.600 |
| **CD** | 1 | 9.966 | 9.966 | 3.24 | 0.214 |
| **3 way interactions** | 4 | 52.954 | 13.238 | 4.30 | 0.198 |
| **ABC** | 1 | 36.178 | 36.178 | 11.75 | 0.076 |
| **ABD** | 1 | 5.311 | 5.311 | 1.72 | 0.320 |
| **ACD** | 1 | 5.085 | 5.085 | 1.65 | 0.328 |
| **BCD** | 1 | 6.380 | 6.380 | 2.07 | 0.287 |

*P value < 0.05 statistically significant difference.
