# Supplementary figures and images for: Functionalized SnO2 nanoparticles with gallic acid via green chemical approach for enhanced photocatalytic degradation of citalopram: synthesis, characterization and application to pharmaceutical wastewater treatment
Source: Environ Sci Pollut Res Int. 2022 Aug 15;30(2):4346–58. doi: 10.1007/s11356-022-22447-5 (PMC9376129; doi:10.1007/s11356-022-22447-5)

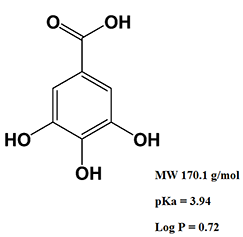

Supplement: Supplementary file 2 — (PNG 11 kb) [file 11356_2022_22447_Fig10_ESM.png]

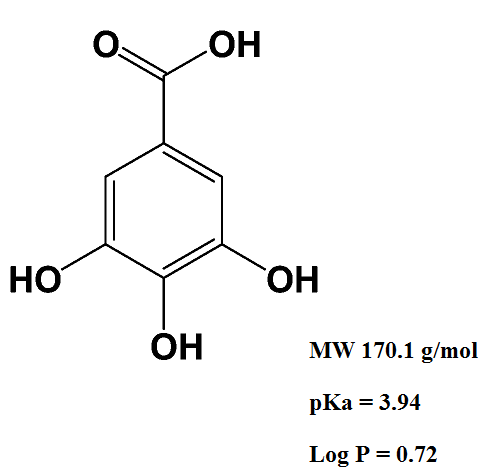

Supplement: Supplementary file 3 — High resolution image (TIF 28 kb) [file 11356_2022_22447_MOESM2_ESM.tif]

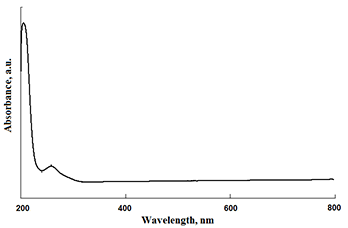

Supplement: Supplementary file 4 — (PNG 8 kb) [file 11356_2022_22447_Fig11_ESM.png]

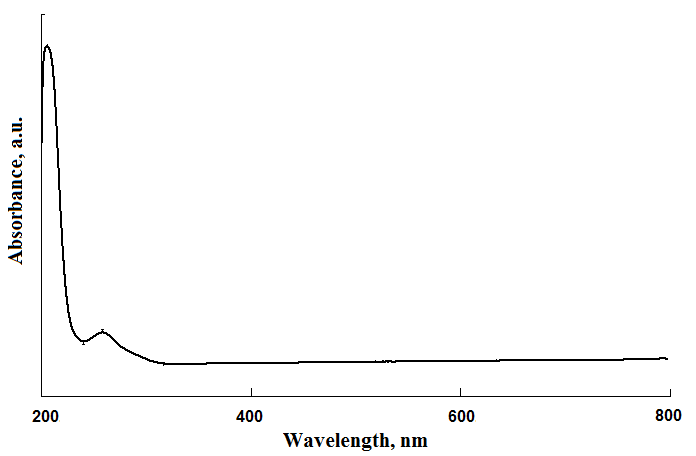

Supplement: Supplementary file 5 — High resolution image (TIF 27 kb) [file 11356_2022_22447_MOESM3_ESM.tif]

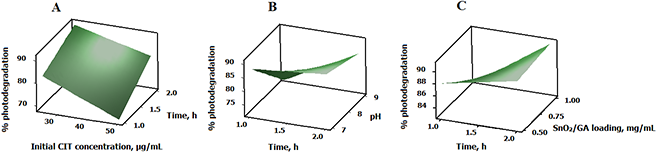

Supplement: Supplementary file 6 — (PNG 41 kb) [file 11356_2022_22447_Fig12_ESM.png]

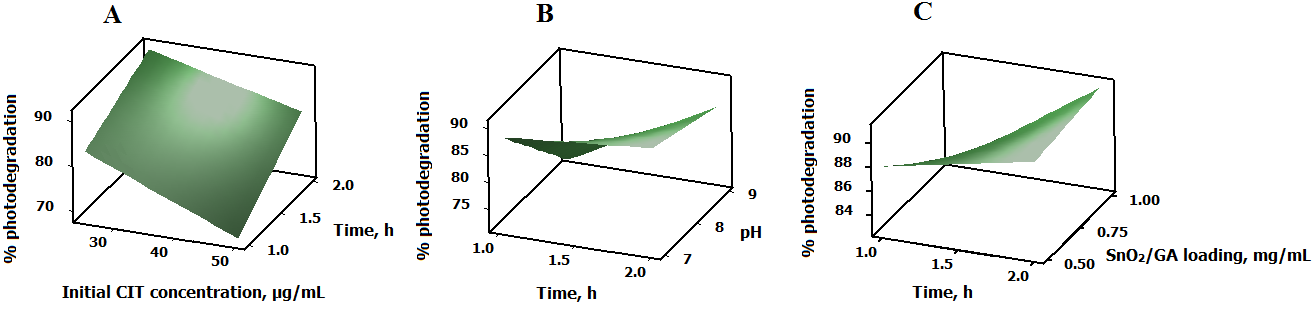

Supplement: Supplementary file 7 — High resolution image (TIF 59 kb) [file 11356_2022_22447_MOESM4_ESM.tif]

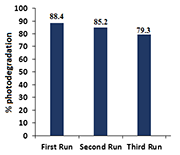

Supplement: Supplementary file 8 — (PNG 9 kb) [file 11356_2022_22447_Fig13_ESM.png]

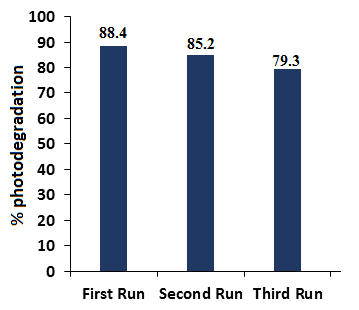

Supplement: Supplementary file 9 — High resolution image (TIF 29 kb) [file 11356_2022_22447_MOESM5_ESM.tif]
